# The SCANVIR^®^ Project: A Success in Hepatitis C Micro-Elimination in Nouvelle-Aquitaine

**DOI:** 10.3390/v18020151

**Published:** 2026-01-23

**Authors:** Sandrine Francois, Gwennaick Villain, Samy Yahiaoui, Christine Silvain, Brigitte Reiller, Paul Carrier, Sophie Alain, Veronique Loustaud-Ratti, Marilyne Debette-Gratien

**Affiliations:** 1 Hepato-Gastroenterology Department, University Hospital Center, 87042 Limoges, France; 2 Hepato-Gastroenterology Unit, University Hospital Center, 86000 Poitiers, France; 3 Risk-Reduction Centers for Drug Users (CAARUD Planterose), Committee for Study and Information on Drugs and Addictions (CEID), 33000 Bordeaux, France; 4 Virology Department, University Hospital Center, 87042 Limoges, France

**Keywords:** hepatitis C micro-elimination, point-of-care molecular testing, liver fibrosis, addiction, precarity, GCS NOVA

## Abstract

The SCANVIR^®^ project is a regional initiative aimed at accelerating the elimination of hepatitis C virus (HCV) by reaching high-risk populations outside traditional healthcare settings. Launched in 2017 in Limoges and later expanded to Poitiers and Bordeaux, the project organized dedicated screening and treatment days in 43 facilities taking care of intravenous drug users, migrants, and prisoners in Nouvelle-Aquitaine. These events involved multidisciplinary teams and advanced diagnostic tools, including rapid tests for HCV, HBV, and HIV; FibroScan^®^ for liver assessment; and GeneXpert^®^ for on-site HCV RNA detection. Patients also received counseling on risk prevention, addiction, psychosocial support, and treatment when needed. Between 2017 and 2024, SCANVIR^®^ screened 1664 patients, with 98.9% accepting FibroScan^®^. Anti-HCV antibodies were detected in 23.4% of participants, among whom 41.5% (*N* = 162) had a replicative profile. Of these, 83% initiated treatment and 80% were cured or were still undergoing therapy. FibroScan^®^ assessments showed advanced fibrosis in 17% of patients, severe fibrosis in 7.2%, and severe steatosis in 18%. By promoting a “Test, Treat, Prevent” strategy, SCANVIR^®^ proved cost-effective in diagnosing and treating individuals distant from care structures, highlighting the value of integrating education and prevention into liver disease screening. SCANVIR^®^ is an officially registered European trademark.

## 1. Introduction

The elimination of hepatitis C (HCV) remains a priority. The prevalence of HCV is around 3% worldwide, and about 210 million people in the world suffer from HCV infection [1]. New direct antiviral agents (DAAs) are available, which are highly effective (sustained virologic response over 95% for all genotypes); well tolerated; and pangenotypic, with a short treatment duration, but most people with HCV remain undiagnosed and untreated. The World Health Organization (WHO) publishes targets that countries must meet to achieve the elimination of HCV by 2030, i.e., diagnosing 90% of people living with HCV and treating 80% [2]. Screening is the cornerstone, treatment is the core step, and prevention is the final step. In 2024, the WHO also published an operational guide on priorities in planning hepatitis B and C screening services. It gathers all guidelines on viral hepatitis screening and helps countries to develop policies and practices for a combined hepatitis B and C screening strategy [3].

In France, estimates show that approximately 79,000 people are infected with HCV [4]. Nevertheless, projections show that France could reach the WHO’s 2030 HCV elimination targets by maintaining current levels of diagnosis and treatment [5]. In order to strengthen our capabilities, it has been proposed to (1) improve access to hepatitis C treatment with new prescribers and a simplified care pathway [6], (2) strengthen local screening through rapid diagnostic tests (RDTs) [7], and (3) create innovative outreach initiatives to reach priority populations who are distant from the healthcare system [8]. This third item is important because it has been clearly established that four out of five HCV patients requiring treatment belong to populations that are distant from health care (drug users, prison populations, psychiatric populations, migrants, and people in precarious situations) [9,10,11]. Therefore, achieving HCV elimination means getting closer to at-risk individuals outside conventional care structures and offering an “all-inclusive” approach with RDTs, HCV RNA detection by point-of-care technology, and immediate DAA prescription.

In 2017, we designed and implemented an innovative concept called the SCANVIR^®^ program to test, treat, and cure people in their own environments. The SCANVIR^®^ concept aimed at removing the barriers to HCV screening and treatment of marginalized patients [12]. We initially tested it in four French departments (Haute-Vienne, Corrèze, Creuse, and Dordogne) and subsequently extended it to a regional level in Nouvelle-Aquitaine. With over 6 million inhabitants and an area of 84,000 km^2^, similar to that of Austria, the Nouvelle-Aquitaine region is the largest in France.

In 2022, SCANVIR^®^ was registered as a project of the Nouvelle Aquitaine Health Cooperation Group (GCS NOVA). This new dimension of the project promotes team interactions, favors resource and activity pooling, and encourages each member to strengthen its position in the region. SCANVIR^®^ is an officially registered European trademark.

Here, we present the results of our outreach screening activities carried out by the three SCANVIR^®^ GSC NOVA teams from 2017 to 2024.

## 2. Materials and Methods

### 2.1. Study Design and Population

**Figure 1:**
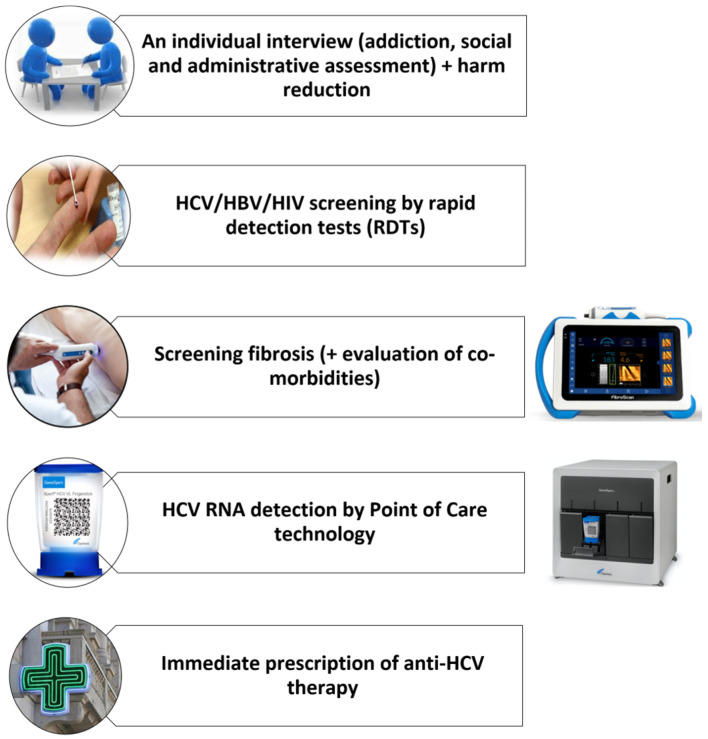
“All-inclusive” strategy in the same temporality.

Starting in May 2017 for the Limoges team and 2019 for the 2 others teams based in Poitiers and Bordeaux, dedicated screening days were organized in each area to bring together a multidisciplinary staff including hepatologists/infectiologists, addictologists, nurses, and social workers. Based on the patients’ profiles, addictology nurses provided regular follow-up (Figure 1). Drug substitution therapies were prescribed if indicated. 

**Figure 2:**
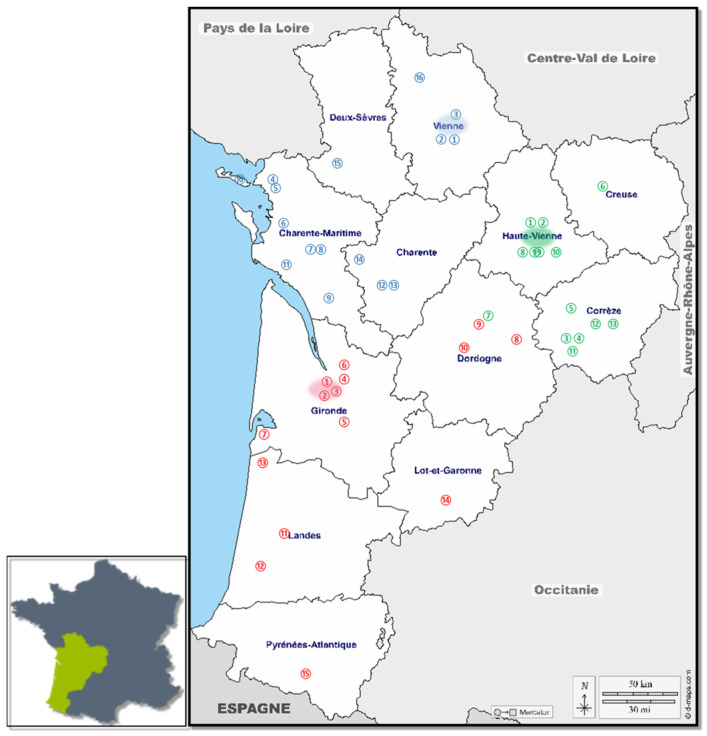
43 structures covering the entire New Aquitaine region: Addiction care centers, Risk-Reduction centers for drug users, Communal centers for social action, Detention facilities, Health centers. In red: structures visited By Bordeaux Team; in blue: structures visited by Poitiers Team; in green: structures visited by Limoges Team.

The three teams, based at the University Hospital of Poitiers, the University Hospital of Limoges, and in a risk-reduction center for drug users (CAARUD) in Bordeaux, were able to visit 43 facilities throughout the Nouvelle-Aquitaine region to offer screening and treatment days. The teams consisted of one or two nurses and a general practitioner or an addiction specialist and/or hepatologist. The Limoges team included a project coordinator, while the Poitiers team was assisted by an administrative assistant responsible for data entry. In addition, staff from the participating institutions were actively involved before, during, and after the SCANVIR sessions. As shown in Figure 2, each team operates in its closest geographical area.

The included patients were ≥18 years old and attended facilities that care for people with risk factors for HIV infection, such as drug treatment centers, harm reduction programs, prisons, community health services, mental health services, and homeless services. Non-opposition to the conduct of the study was collected.

### 2.2. Nature of Care Offered: Screening Tools and Risk Prevention

Liver stiffness measurement:

The stage of liver fibrosis was assessed trough liver stiffness measurement (LSM) by FibroScan^®^ (FibroScan 430 Mini, ECHOSENS™). Since 2023, the Limoges team has been using the new version of FibroScan^®^ to measure steatosis using the controlled attenuation parameter (CAP) (FibroScan 430 Mini+ with FS 4.1.3 software, ECHOSENS™). Results of LSM are expressed in kilopascals (kPa) and were validated by 10 consecutive inter-costal measurements and an IQR/median < 30%. Results of CAP are expressed in decibels per meter (dB/m). CAP commonly ranges between 100 and 400 dB/m, and an IQR/median of <30% was considered reliable for the CAP score. The presence of massive liver steatosis was defined by a median CAP ≥ 280 dB/m [13]. Concerning FibroScan^®^, different LSM thresholds were considered: LSM < 8 kPa to rule out compensated advanced chronic liver disease (cACLD), LSM ≥ 12–15 kPa to confirm cACLD, and LSM ≥ 20–25 kPa to define clinically significant portal hypertension [14]. All patients with LSM > 12 kPa were referred to the specialist for follow-up, even if the RDTs were negative. Patients with LSM ≥ 8 and ≤12 kPa were either referred to specialized care or offered an annual LSM follow-up.

Screening for HCV, HBV, and HIV:

Different RDTs were used to screen HCV, HBV, and HIV infections (Table 1). All are certified to be used on capillary blood (European Conformity, CE). Not all three tests were mandatory, and participants could choose one of the three tests.

HCV RNA detection by RT-PCR:

HCV RNA must be detected when the rapid HCV screening test is positive. As soon as the GeneXpert technology became available in November 2019, a blood sample was taken for RT-PCR testing, and the specialist offered a consultation immediately or within a week to communicate the results to the patients and begin their treatment if necessary. HCV RT-PCR tests were carried out in situ using a GeneXpert^®^ device (Xpert^®^ HCV VL Fingerstick, Cepheid, Sunnyvale, CA, USA), with a 100 µL drop of blood collected by micropuncture. The result was obtained within 59 min on site, further reducing the period of care.

For the Limoges and Poitiers teams, this dedicated GeneXpert^®^ device was under the responsibility of the virology department at Limoges University Hospital. An agreement was signed between the virology and hepato-gastroenterology departments. The staff who performed testing during SCANVIR^®^ visits were trained by virologists and carried out the analyses under their supervision. A calibrated positive control (PC) was tested before each outing [15] Cepheid^®^ (Cepheid, USA) provided maintenance of the GeneXpert^®^ device and staff training for the Bordeaux team.

The limit of detection provided by the manufacturer is 22 IU/mL for the HCV genotype 1a and 35 IU/mL for genotype 6e (WHO international reference standard [3]), and all HCV genotypes (1 to 6) are detected with a range of 100 to 10^8^ IU/mL.

## 3. Results

### 3.1. Participants

Between 2017 and June 2024, 43 different structures, such as addiction care centers, risk-reduction centers for drug users, communal centers for social action, detention facilities, and health centers, were visited (Figure 2).

**Figure 3:**
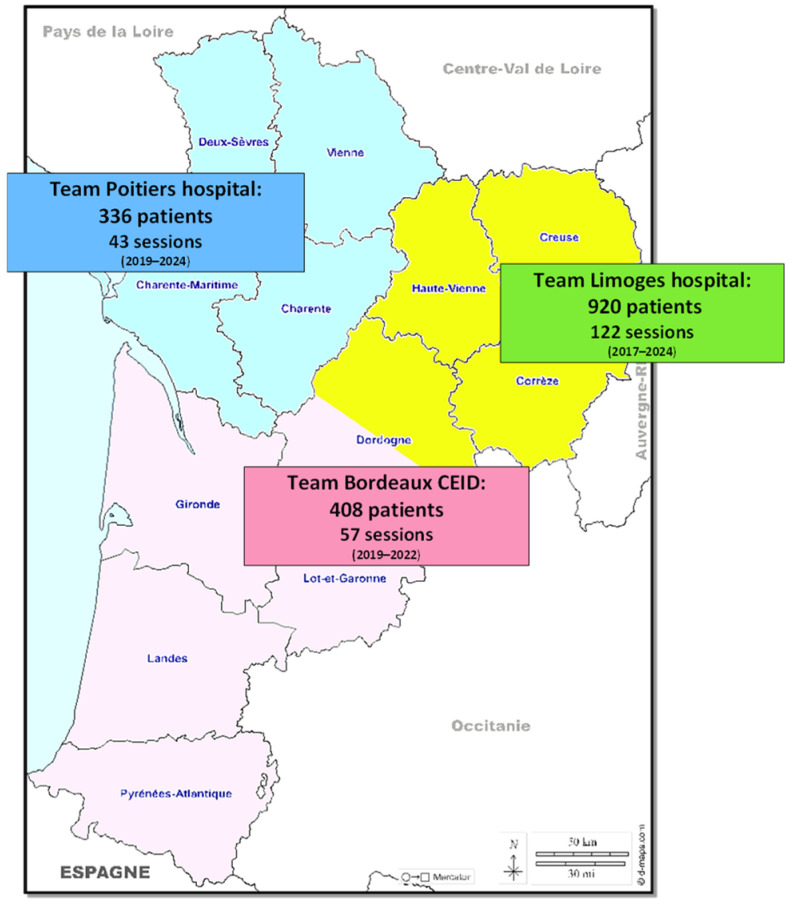
Numbers of patients screened by each team in the Nouvelle-Aquitaine region.

In total, 222 dedicated days were scheduled, and 1664 patients were screened (Figure 3), corresponding to 2164 RDTs and 1604 LSMs performed. The Limoges team screened 920 participants, with a median age of 46 years old and a sex ratio of 2.8.

**Figure 4:**
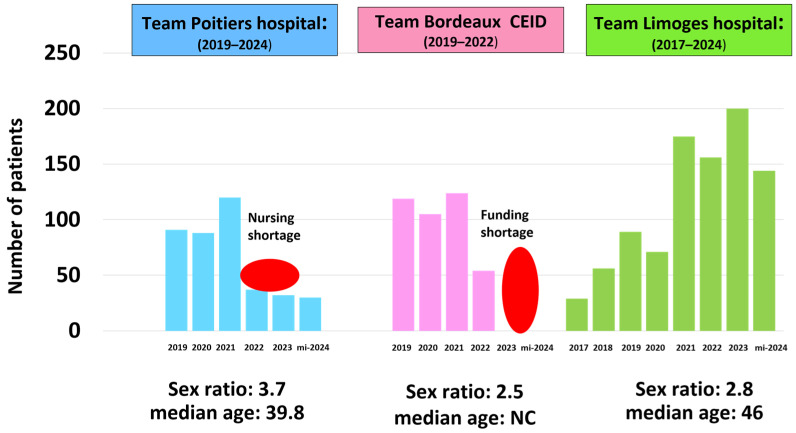
Numbers of patients screened per year by each team.

The SCANVIR^®^ concept was implemented retrospectively for the two other teams. Between 2019 and June 2024, the Poitiers team screened 336 patients during 43 sessions, with a median age of 39.8 years old and a sex ratio of 3.7. Due to a shortage of nursing staff, the number of screenings decreased from 2022 to 2024. From 2019 to 2022, the Bordeaux team screened 408 patients in 57 sessions, with a sex ratio of 2.5. The age of participants was not collected. For this team, no screening was performed from 2023 to June 2024 due to funding interruption (Figure 4). Of note, new funding enabled screening to resume in 2024.

### 3.2. Consumption of Addictive Substances

**Figure 5:**
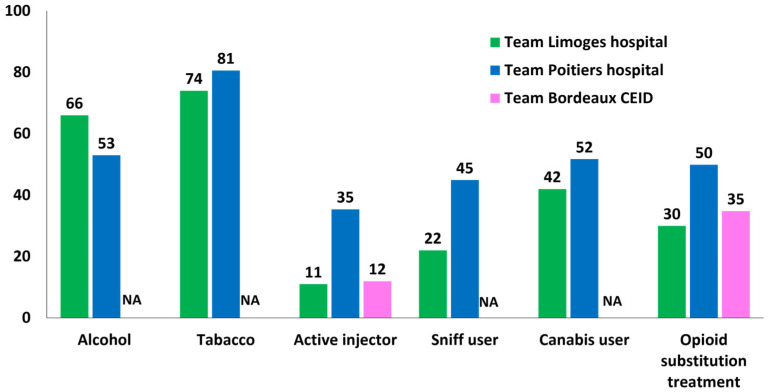
Proportion of patients by substance consumption. NA: data not available.

On average, 19.3% were active drug injectors, and 38.3% had a substitution opioid treatment. A proportion of 59.5% declared excessive alcohol consumption, 77.3% smoked, 33.5% snorted drugs, and 47% consumed cannabis (Figure 5). The Poitiers team took care of the largest number of patients who injected or snorted drugs or used cannabis, as well as the largest number of patients under opioid substitution treatment (Figure 5).

### 3.3. HCV, HBV, and HIV Screening

**Figure 6:**
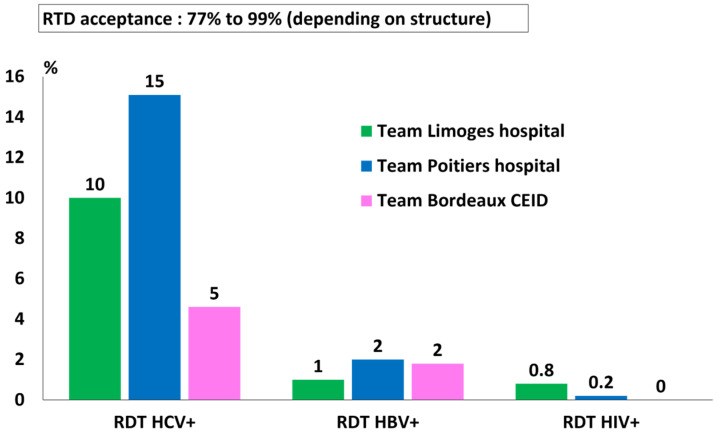
Proportion of positive RDT results per team.

**Figure 7:**
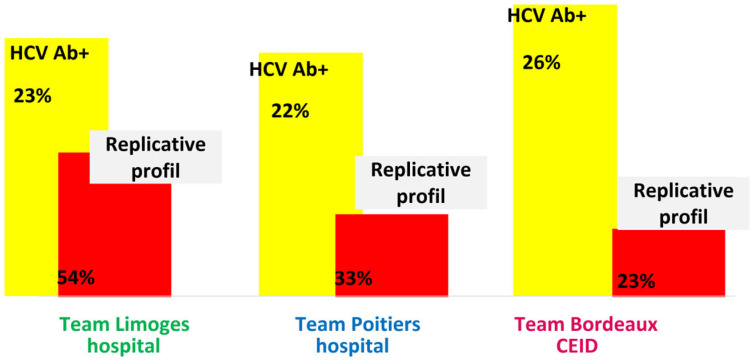
Proportions of positive HCV serostatus and viremia (replicative status) per team.

On average, across the region, 10% of HCV RDTs, 1.6% of HBV RDTs, and 0.3% of HIV RDTs were positive (Figure 6). RDT acceptance was 77% to 99%, depending on the structure. The percentage of HCV RDT+ differed considerably depending on the geographical zone because not all teams systematically re-tested with RDT patients who already knew their HCV Ab+ status. However, the prevalence of HCV antibodies, calculated by summing the results of positive rapid tests and patients known to be HCV-positive but not tested, was comparable in the three geographical areas, with an average of 23.4%, corresponding to 390 patients (Figure 7).

All of them benefited from HCV RNA detection via the point-of-care GeneXpert^®^ device or remote laboratory testing before 2019. Results showed that the replicative profiles of HCV Ab+ patients were heterogeneous, ranging from 22.6% for the Bordeaux team to 54% for the Limoges team. Overall, 162 patients with replicative HCV infection were identified (Figure 7).

All patients infected with HIV were aware of their status and were receiving antiretroviral treatment. Patients carrying HBsAg were referred to hepatologists or infectiologists in a hospital or private practice (networks of partner physicians identified by the organization).

### 3.4. Fibrosis and Steatosis Evaluation

**Figure 8:**
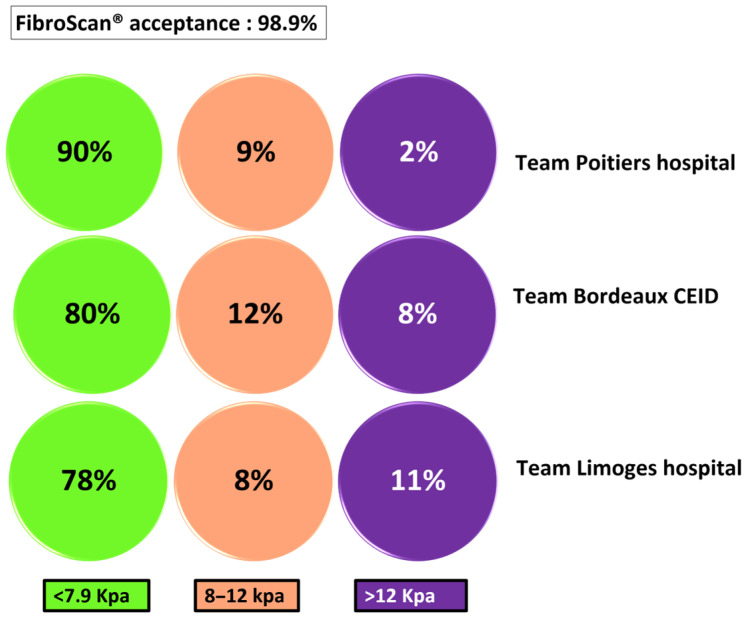
Proportion of patients with minimal fibrosis, moderate fibrosis, and advanced fibrosis/cirrhosis per FibroScan.

LSM by FibroScan^®^ was extremely well accepted, with a compliance rate of 99%. A proportion of 17% of patients were suspected to have advanced fibrosis (elasticity > 8 kPa), and severe fibrosis (elasticity > 12 kPa) was very likely in 7.2% of the participants (Figure 8*).* The highest level of fibrosis was reported in the region screened by the Limoges team, where 11% of participants had severe fibrosis. Alcohol consumption was also the highest in this area (Figure 5 and Figure 8). The lowest level of fibrosis was recorded in the Bordeaux region, with 2% severe fibrosis (Figure 8).

**Figure 9:**
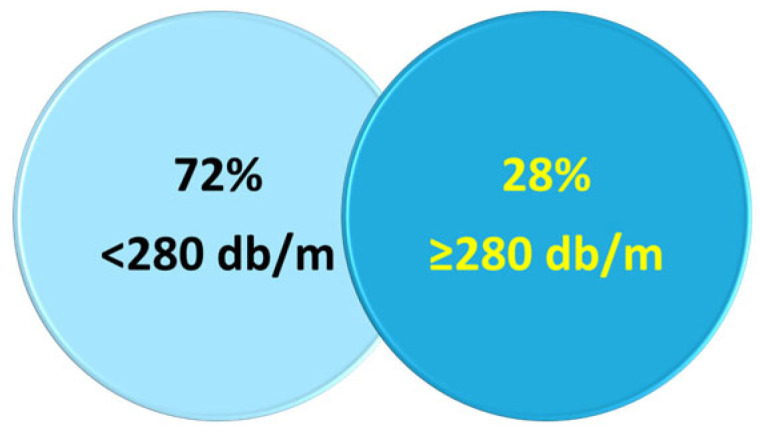
Proportion of patients with and without severe steatosis per CAP assessment.

Among the 234 patients for whom CAP was available, 28% had severe steatosis (CAP > 280 dB/m) (Figure 9). In cases of advanced fibrosis (>8 kPa), participants were encouraged to contact their general practitioner. In cases of severe fibrosis (>12 kPa), comorbidities were assessed, and a rapid consultation with a hepatologist in the patient’s screening area was offered. Prevention messages were emphasized. In the absence of a general practitioner, the facility took charge of the patients to reassess their social and medical situation.

### 3.5. Treatment

**Figure 10:**
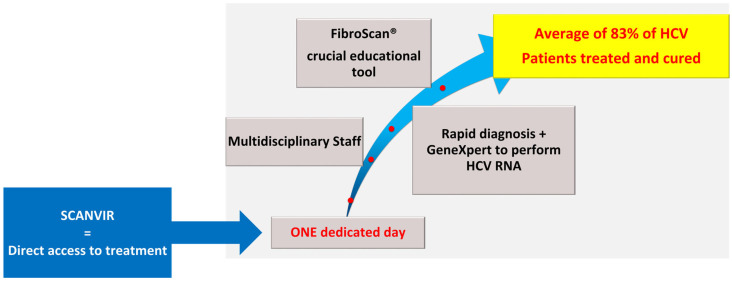
SCANVIR program summary.

When the replicative profile for HCV infection was confirmed by GeneXpert, treatment with DAAs was offered immediately by the attending physician. Nurses provided therapeutic education. If necessary, staff from the facilities supported the patients during the treatment (health insurance, delivery to the pharmacy, food, accommodation, etc.). In our study, an average of 83% (*N* = 135) of HCV patients were treated and cured with DAAs (82% to 85%). The main reasons why 10% of patients were not cured were death, loss to follow-up, refusal to be treated, lack of social protection, and delay of treatment for other reasons (Figure 10).

## 4. Discussion

To eliminate hepatitis C, it has been clearly demonstrated that it is important to focus on screening populations with high HCV prevalence, such as intravenous and/or nasal drug users, people living in precarious conditions, migrants, etc. [16]. As these individuals are often isolated from healthcare facilities, it is necessary to develop “outreach” programs. The SCANVIR^®^ project stands out for its innovative nature, offering days specifically dedicated to the screening of viral hepatitis B and C and HIV and the assessment of liver fibrosis within various structures. Thanks to the collaboration of three multidisciplinary teams based in Limoges, Poitiers, and Bordeaux, the program covers a large part of the Nouvelle-Aquitaine region—the largest in France.

Implementation of such actions represents a significant cost, which makes institutional financial support essential. As shown here, the lack of funding or staff compromised screening for some years (Figure 4). In this regard, the SCANVIR^®^ project benefits from the support of the Nouvelle-Aquitaine Regional Health Agency (ARS), the University Hospitals in Limoges and Poitiers, and the CEID in Bordeaux.

Using portable diagnostic tools (RTDs, FibroScan^®^, and GeneXpert^®^), we screened 1664 people across 43 facilities in this area. Faced with the diversity of partner facilities—prisons, social action centers, specialized addiction treatment centers, and psychiatric services—the SCANVIR^®^ teams demonstrated a great adaptive capacity. The patient population was predominantly male, with one-fifth using injectable drugs and one-third using nasal drugs. We observed a fairly sharp decline in the use of injectable drugs compared to published data, but conversely, alcohol and tobacco consumption remained very high [17,18]. The use of opioid substitution treatments drastically increased compared with a study conducted between 2018 and 2023 in 26 medical departments specialized in addiction treatment (38.3% vs. 7%) [18]. However, the population studied by Chevaliez et al. was different and came mainly from addiction and risk-reduction centers.

Screening remains highly cost-effective in settings with a high prevalence of hepatitis C virus [19,20]. In our study, it also appeared to be cost-effective; however, this project did not include a formal cost-effectiveness analysis. The average prevalence of HCV seropositivity was 23.6%, with a relatively uniform rate across the three areas. The fact that we visited more varied facilities could explain the difference relative to the prevalence reported by Chevaliez et al. (43.6% vs. 23.6%). The prevalence of hepatitis B (HBsAg+) and HIV remained very low, as reported by Chevaliez et al. [18].

On average, nearly half of the patients seropositive for HCV showed active viral replication, with some disparity across the three territories, ranging from 23% to 54%. This is comparable to data reported with a similar level of replicative profile [17,18].

We had a confirmed cure rate of between 82% and 85% for replicative patients who received DAA therapy. Once again, these results are consistent with those of two other studies conducted in France [17,18]. Since 2019, GeneXpert has played a decisive role in enabling immediate treatment initiation. The few patients who did not receive treatment had either stopped attending appointments before starting treatment (so-called “volatile” patients who frequently change region or place of residence) or actively refused any form of therapy (loss of trust in the medical system, i.e., paranoia). This situation requires strategies from the staff at the facilities to restore trust. In practice, social workers within these institutions are key, both upstream of our interventions and following the screening sessions, to mobilize participants, support access to social entitlements, and facilitate the coordination of medical care pathways.

In all the structures, the SCANVIR^®^ project also contributed to harm reduction. However, although hepatitis C screening must be offered to all incarcerated individuals, detention centers have different practices and face financial, linguistic, and medical obstacles [21]. In France, the law on the modernization of the healthcare system of 26 January 2016 states that the policy for risk and harm reduction also applies to prisoners, according to procedures adapted to the prison environment [22]. However, no decree implementing the law specifies these adapted procedures to date.

Our study highlights a rate of advanced or severe liver fibrosis (LSM > 8 kPa) reaching up to 20%, as well as a significant proportion of severe hepatic steatosis (CAP > 280 dB/m) observed in 28% of participants. Some studies have shown that the prevalence of advanced fibrosis and cirrhosis may be high in vulnerable populations identified as suffering from MetALD and ALD [23]. In our study, liver damage was associated with various factors, including excessive alcohol consumption, a sedentary lifestyle, nutritional imbalances, and prolonged use of psychotropic drugs. These patients were immediately referred to the specialized care pathway appropriate to their clinical situation.

For people with moderate liver damage, our interventions played a decisive role in terms of education and prevention. Indeed, the use of FibroScan^®^ as a medical visualization and communication tool favored individual awareness. This awareness can be the starting point for change, whether it be alcohol withdrawal, a change in diet, increased physical activity, or therapeutic adjustment when hepatotoxic treatments are identified.

Currently, the SCANVIR^®^ project is continuing its outreach efforts, which aim to identify patients who have not been diagnosed with HCV infection and locate potential hot spots of transmission. Harm reduction remains a major component in this fight. Combined use of opioid agonist therapy with needle and syringe programs is associated with a 74% reduction in risk of HCV infection [24]. However, access to preventive services for people who inject drugs in prison facilities is extremely limited in Europe [25]. Therefore, our actions are very important in this setting, as well as in psychiatric settings, which have a high prevalence of HCV [26].

Furthermore, it is essential not to neglect vaccination against hepatitis B. A particular challenge lies in taking care of vulnerable populations and migrants, who are often poorly informed. This is in line with the objectives of the 2018–2028 Regional Health Project [27] and the Global Health Sector Strategies on HIV, viral hepatitis and sexually transmitted infections 2022–2030 [2]. Finally, telemedicine could offer an additional opportunity to provide remote care [28,29], in addition to involving advanced practice nurses, who play a key role in supporting and monitoring patients and implementing treatments.

## 5. Conclusions

Our study confirms the ongoing widespread circulation of hepatitis C and highlights the necessity of continued eradication initiatives. The SCANVIR^®^ project is an innovative pathway promoting the “Test, Treat, and Prevent” strategy to eliminate HCV. Created in 2017 in Limoges, the SCANVIR^®^ concept has been extended to the whole of the Nouvelle–Aquitaine region, with two other teams based in Poitiers and Bordeaux. Furthermore, as members of GCS NOVA, we can consolidate our practices and share our results. This concept makes diagnostic testing cost-effective for high-risk patients far from healthcare facilities, with an HCV cure rate of up to 85%. In addition to the educational role played by FibroScan^®^ in “catching” patients who refuse RDTs, we highlight the importance of screening for liver fibrosis and steatosis, consequences of increasingly frequent alcoholic and metabolic disorders in this population.

## Figures and Tables

**Table 1:** RDTs used by the three teams.

Team	HCV Ab Detection	HBV HBsAg Detection	HIV Ab Detection
Bordeaux	First Response^®^ HCV card Test (Biosynex SA, Ill-kirch-Graffenstaden, France)	First Response^®^ HBsAg card Test (Biosynex SA, Illkirch-Graffenstaden, France)	EXACTO PRO^®^ TEST HIV (Biosynex SA, Illkirch-Graffenstaden, France)
Poitiers	TOYO^®^ Anti VHC (Nephrotek, Boulogne, France)	TOYO^®^ HBSAg (Nephrotek, Boulogne, France)	INSTI^®^ VIH (bioLytical, Richmond, BC, Canada)
Limoges	TOYO^®^ Anti VHC (Nephrotek, Boulogne, France)	First Response^®^ HBsAg card Test (Biosynex SA, Illkirch-Graffenstaden, France)	Determine™ HIV Ultra (Abbott, Lake Bluff, IL, USA) *

* *Simultaneous detection of P24 antigen and anti-HIV antibodies*.

## Data Availability

The original contributions presented in this study are included in the article. Further inquiries can be directed to the corresponding author.

## Abbreviations

The following abbreviations are used in this manuscript:

ALD	Alcoholic liver disease
CAP	Controlled attenuation parameter
CAARUD	Risk-reduction centers for drug users
HIV	Human immunodeficiency virus
HBV	Hepatitis B virus
HCV	Hepatitis C virus
DAA	Direct antiviral-acting agents
dB/m	Decibels per meter
kPa	Kilopascals
cACLD	Compensated advanced chronic liver disease
RT-PCR	Reverse transcriptase polymerase chain reaction
MetALD	Metabolic dysfunction and alcohol-related liver disease
LSM	Liver stiffness measurement
RDTs	Rapid diagnostic tests
RNA	Ribonucleic acid
WHO	World Health Organization
